# Emerging systemic therapy options beyond CDK4/6 inhibitors for hormone receptor-positive HER2-negative advanced breast cancer

**DOI:** 10.1038/s41523-023-00578-3

**Published:** 2023-09-08

**Authors:** Jun Ma, Jack Junjie Chan, Ching Han Toh, Yoon-Sim Yap

**Affiliations:** 1 Division of Medical Oncology, National Cancer Centre Singapore, 30 Hospital Boulevard, Singapore, 168583 Singapore; 2 Oncology Academic Clinical Programme, Duke-NUS Medical School, 8 College Road, Singapore, 169857 Singapore

**Keywords:** Breast cancer, Target identification, Tumour biomarkers, Cancer therapeutic resistance

## Abstract

Endocrine therapy (ET) with cyclin-dependent kinase 4/6 inhibitor (CDK4/6i) is currently the standard first-line treatment for most patients with hormone receptor (HR) positive, human epidermal growth factor receptor (HER2) negative advanced breast cancer. However, resistance to ET and CDK4/6i inevitably ensues. The optimal post-progression treatment regimens and their sequencing continue to evolve in the rapidly changing treatment landscape. In this review, we summarize the mechanisms of resistance to ET and CDK4/6i, which can be broadly classified as alterations affecting cell cycle mediators and activation of alternative signaling pathways. Recent clinical trials have been directed at the targets and pathways implicated, including estrogen and androgen receptors, PI3K/AKT/mTOR and MAPK pathways, tyrosine kinase receptors such as FGFR and HER2, homologous recombination repair pathway, other components of the cell cycle and cell death. We describe the findings from these clinical trials using small molecule inhibitors, antibody–drug conjugates and immunotherapy, providing insights into how these novel strategies may circumvent treatment resistance, and discuss how some have not translated into clinical benefit. The challenges posed by tumor heterogeneity, adaptive rewiring of signaling pathways and dose-limiting toxicities underscore the need to elucidate the latest tumor biology in each patient, and develop treatments with improved therapeutic index in the era of precision medicine.

## Introduction

The hormone receptor-positive (HR + ) and human epidermal growth factor receptor-2 negative (HER2-) subtype comprises ~68% of all breast cancers (BCs)^1^. Endocrine therapy (ET) with cyclin-dependent kinase 4/6 inhibitor (CDK4/6i) is currently the standard treatment for HR + /HER2− advanced BC (ABC)^2,3^, with the demonstration of overall survival (OS) benefit in several trials^4–7^. However, treatment resistance inevitably ensues, and the optimal management after prior CDK4/6i plus ET continues to be refined, especially with the increasing use of adjuvant CDK4/6i^8^, as well as adjuvant poly ADP-ribose polymerase inhibitors (PARPi)^9^, changing the profile of patients with recurrent metastatic disease. Currently, there is a lack of data on the tumor biology and the treatment strategies after relapse in these patients. Tumor and/or liquid biopsy upon relapse or progression to determine the latest HR and HER2 status as well as genomic profile may provide insight into the underlying resistance mechanisms, and allow an individualized approach to treatment. New therapeutic targets may be discovered from profiling the resistant metastatic lesions, which harbor acquired alterations absent in the primary tumor^10–12^.

There is considerable heterogeneity among the HR + /HER2− BCs. With gene expression profiling, distinct intrinsic subtypes (IS) of BCs may be identified, which predict for differing prognosis and response to endocrine therapy. Despite being HR+ by immunohistochemistry (IHC), luminal B tumors have a higher expression of proliferation/cell cycle-related genes, and predict a worse prognosis compared to luminal A tumors^13,14^. IS classification also predicts for response to treatment, with a shorter time to progression on ET with CDK4/6i in luminal B, HER2 enriched, and basal subtypes compared to luminal A tumors^15^. Furthermore, upon disease progression, conversion of IS from a luminal type to a nonluminal type can occur, leading to a more aggressive endocrine-resistant biology^13^.

**Fig. 1:**
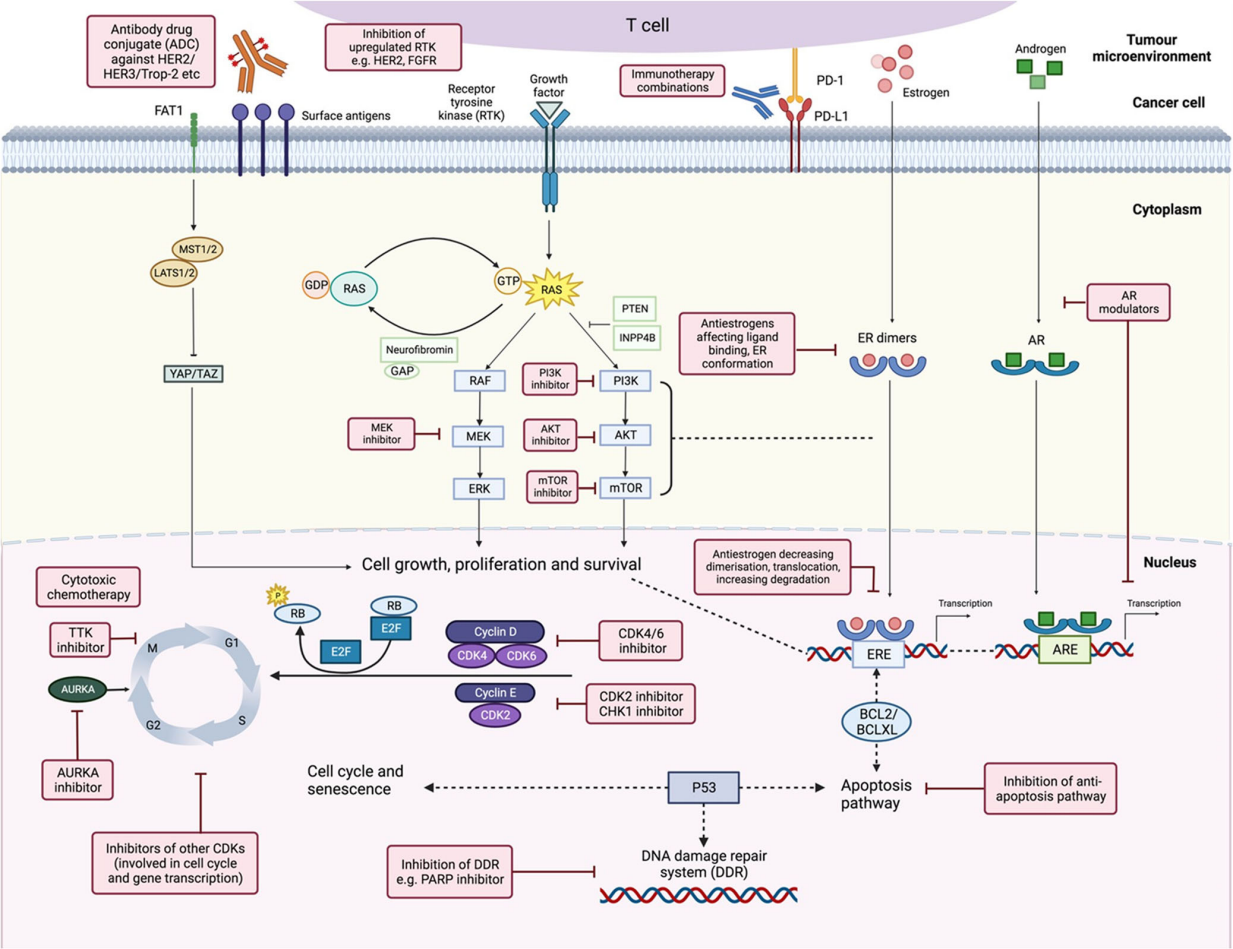
Oncogenic signaling pathways in HR + /HER2− ABC with potential therapeutic strategies post CDK4/6 inhibitors. The potential treatment strategies are shown in pink boxes, while crosstalk is indicated by dotted lines (figure created with BioRender.com). AKT V-akt murine thymoma viral oncogene homolog 1, AR androgen receptor, ARE androgen response element, AURKA aurora kinase A, BCL2 B-cell lymphoma 2, BCLXL B-cell lymphoma-extra large, CDK cyclin-dependent kinase, CHK checkpoint kinase, ER estrogen receptor, ERE estrogen response element, ERK extracellular signal‑regulated kinase, FGFR fibroblast growth factor receptor, GAP GTPase-activating protein, GDP guanosine diphosphate, GTP guanosine triphosphate, HER human epidermal growth factor receptor, INPP4B inositol polyphosphate 4-phosphatase type II, LATS large tumor suppressor kinase, MEK mitogen‑activated protein kinase, MST mammalian STE20-like kinase, mTOR mammalian target of rapamycin, PARP poly(ADP-ribose) polymerase, PD-1 programmed cell death protein 1, PD-L1 programmed death-ligand 1, PI3K phosphatidylinositol-3-kinase, PTEN phosphatase and tensin homolog, RAS rat sarcoma viral oncogene homolog, RAF rapidly accelerated fibrosarcoma kinase, RB retinoblastoma tumor suppressor, TAZ transcriptional co-activator with PDZ-binding motif, Trop-2 trophoblast cell-surface antigen 2, TTK spindle assembly checkpoint kinase, YAP Yes-associated protein.

Mechanisms of resistance to CDK4/6 blockade can be broadly categorized as aberrations affecting cell cycle progression and activation of other signaling pathways as described previously^12,16^ (Fig. 1). The alterations which affect cell cycle mediators include loss-of-function alterations in *RB1* and upregulation of CDK6, cyclin E1/E2, and Aurora kinase A. Activation of the phosphoinositide 3-kinase (PI3K)/protein kinase B (AKT)/mammalian target of rapamycin (mTOR) and the mitogen-activated protein kinase (MAPK) pathways can be effected through activating mutations of oncogenes or loss of function of tumor suppressors, while activating mutations or amplification involving other growth factor receptor genes such as *ERBB2* (HER2) and *FGFR* (fibroblast growth factor receptor)^16–18^ can lead to signaling through alternative oncogenic pathways. Primary or secondary resistance to the ET backbone can occur concurrently^18,19^. Given the differential properties of the three CDK4/6is which may influence the mode of action, the underlying mechanisms of resistance may potentially vary. Data from such research is awaited.

**Fig. 2:**
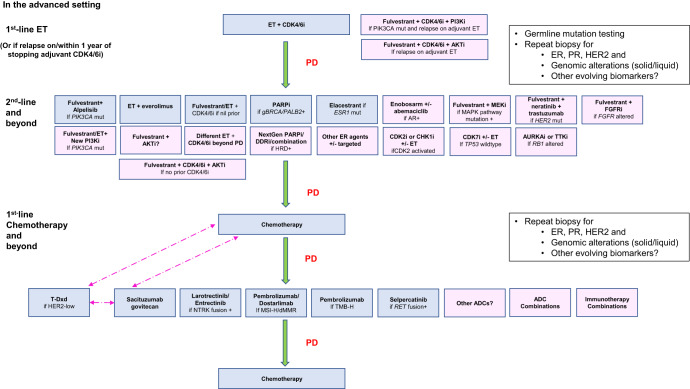
Current and future treatment paradigms for HR + /HER2− ABC. Blue boxes indicate current treatment options based on FDA-approved drugs and the latest NCCN^162^, ESMO, ASCO guidelines^2,3^. Pink boxes indicate the potential therapeutic strategies in the future, which are currently still investigational. Dotted pink arrows indicate the possibility of switching between treatments, or change in sequence of the treatment options, pending further data. ADC antibody–drug conjugate, AR androgen receptor, DDRi DNA damage repair inhibitor, dMMR deficient mismatch repair, ET endocrine therapy, *ESR1* estrogen receptor 1 gene, *gBRCA* mut germline *BRCA1*/*BRCA2* mutation (now termed variant), HRD homologous recombination deficiency, MAPK mitogen-activated protein kinase, MSI-H microsatellite instability high, NTRK neutotrophic tyrosine receptor kinase, *PALB2* mut partner and localizer of BRCA2 mutation, *RET* rearranged during transfection, T-DXd trastuzumab deruxtecan, TMB-H tumor mutational burden high, *TP53* transformation-related protein 53.

While mechanisms involving other hallmarks of cancer such as deregulation of cellular metabolism, epigenetic reprogramming, pro-tumorigenic inflammation, and microenvironment^18,20^, may be implicated, this review will focus on the therapeutic strategies with reported clinical trial activity that have the potential to transform our treatment paradigm over the next decade (Figs. 1 and 2). For the various strategies, we describe the preclinical and clinical rationale, summarize the efficacy demonstrated in clinical trials, highlight the challenges and discuss the implications for patient management with future directions.

## Prospective therapeutic strategies post cdk4/6 inhibition

### Targeting the estrogen receptor (ER) pathway

Estrogen receptor 1 (*ESR1*) activating mutations at the ligand binding domain of ER are rare in untreated primary BCs, but are typically enriched after exposure to aromatase inhibitors (AIs) with a prevalence of ~20–40% in pretreated ABC^21–24^, promoting estrogen-independent constitutional activation and downstream transcription of ER-controlled genes^25^. Other effects of *ESR1* mutations include the induction of metabolic alterations^26^, distinct cistromes, and transcriptional changes to stimulate growth and metastases^27^.

Tamoxifen is a SERM (selective estrogen receptor modulator) which competes with estrogen in binding to ER and partially inhibits mutant ERα transcription. However, more potent ER antagonists may be more efficacious. Preclinical studies showed that lasofoxifene, a potent antagonist of both wild-type and mutant ER^28^, was effective in reducing tumor growth in an endocrine-resistant *ESR1*-mutated xenograft model^29^. However, ELAINE 1, an open-label phase II trial of patients with HR + /HER2− *ESR1*-mutated ABC with progression after at least 12 months of AI and CDK4/6i, failed to show a statistically significant improvement in progression free survival (PFS) of lasofoxifene over fulvestrant^30^. Otherwise, in the single-arm phase II ELAINE 2 trial, among 29 women with HR + /HER2− *ESR1*-mutated metastatic BC (MBC) whose disease progressed after 1–2 prior lines of ET with or without CDK4/6i, lasofoxifene plus abemaciclib was well-tolerated and demonstrated activity with median PFS (mPFS) of 13.9 months and objective response rate (ORR) of 33.3%^31^.

Fulvestrant, a pure ER antagonist and the only clinically approved SERD (selective estrogen receptor degrader) until recently, not only reduces ER dimerization and transcription, but also induces ER degradation via a proteasome-dependent system^25^. Fulvestrant is superior to AI in the presence of *ESR1* mutations^32^, but higher drug levels are required for optimal activity, especially in Y537S-mutant cells^10,25^. Fulvestrant is poorly soluble, requiring administration via intramuscular injections and limiting the volume or dose that can be delivered. The standard high dose of 500 mg fulvestrant failed to completely abolish ER availability in tumors of 38% of patients in a [^18^F]fluoroestradiol (FES) positron emission tomography/computed tomography (PET/CT) study^33^. Hence there is great interest in developing oral SERDs for which the dose may be escalated to optimize antitumor activity, as well as complete ER antagonists (CERANs), selective ER covalent antagonists (SERCAs) and proteolysis targeting chimeras (PROTACs). PROTACs facilitate proteosomal degradation of target proteins via ubiquitination^34^, while SERCAs covalently inactivate both wild-type and mutant ER by inducing conformational changes in the ER, reducing binding of co-activators^35^. These agents are being tested in ongoing trials, including combination trials with CDK4/6 or PI3K/AKT/mTOR inhibition (Table 1).

**Table 1:** Selected trials of novel estrogen receptor targeting drugs recruiting patients with HR + HER2− ABC (last updated August 2023).

Clinicaltrials.gov identifier	Phase	Drug	Drug class	Treatment arm(s)	Study population	Study status
NCT04791384	Ib/II	Elacestrant	SERD	Elacestrant + abemaciclib	Postmenopausal, HR+/HER2− MBC with brain metastasis, up to 2 lines of prior CT	Recruiting
NCT05618613 (ELONA)	Ib/II	Elacestrant	SERD	Elacestrant + onapristone	Previously treated HR+/HER2− ABC with prior ET + CDK4/6i	Active, not recruiting
NCT04546009 (PersevERA)	III	Giredestrant (GDC-9545)	SERD	Giredestrant + palbociclib Letrozole + palbociclib	Previously untreated HR+/HER2− ABC	Active, not recruiting
NCT04802759	Ib/II	Giredestrant	SERD	Giredestrant Giredestrant + abemaciclib Giredestrant + ipasertib Giredestrant + inavolisib Giredestrant + ribociclib Giredestrant + everolimus Giredestrant + samuraciclib Giredestrant + atezolizumab Giredestrant + abemaciclib + atezolizumab	Cohort 1: Advanced/MBC HR+/HER2− with progression on 1-2L ET + CDK4/6i	Recruiting
NCT03616587 (SERENA-1)	I	Camizestrant (AZD9833)	SERD	Camizestrant Camizestrant + palbociclib Camizestrant + everolimus Camizestrant + abemaciclib Camizestrant + capivasertib Camizestrant + ribociclib Camizestrant + anastrozole	Endocrine-resistant HR+/HER2− ABC	Recruiting
NCT04711252 (SERENA-4)	III	Camizestrant	SERD	Camizestrant + palbociclib Anastrozole + palbociclib	Untreated HR+/HER2− ABC	Recruiting
NCT04964934 (SERENA-6)	III	Camizestrant	SERD	Camizestrant + CDK4/6i AI + CDK4/6i	HR+/HER2− ABC on 1L AI + CDK4/6i with ctDNA detected *ESR1* mutation	Recruiting
NCT04188548 (EMBER)	Ia/Ib	Imlunestrant (LY3484356)	SERD	Imlunestrant Imlunestrant + abemaciclib +/− AI Imlunestrant + everolimus Imlunestrant + alpelisib	Part A up to 1L therapy HR+/HER2− ABC, no CDK4/6i Part B HR+/HER2 ABC with prior CDK4/6i	Active, not recruiting
NCT04975308 (EMBER-3)	III	Imlunestrant	SERD	Imlunestrant Imlunestrant + abemaciclib Physician’s choice ET (fulvestrant/exemestane)	Postmenopausal HR+/HER2− ABC with prior AI ± CDK4/6i	Recruiting
NCT04568902	I	H3B-6545	SERCA	H3B-6545	HR+/HER2− MBC with at least 2 prior ET, or 1 prior ET and 1 prior CT, or 1 prior ET + CDK4/6i	Active, not recruiting
NCT04288089	I	H3B-6545	SERCA	H3B-6545 + palbociclib	Previously treated locally advanced/MBC HR+/HER2−	Active, not recruiting
NCT04505826	I/II	OP-1250	CERAN	OP-1250	Previously treated locally advanced/MBC HR+/HER2−	Active, not recruiting
NCT05266105	I	OP-1250	CERAN	OP-1250 + palbociclib	HR+/HER2− ABC	Recruiting
NCT05508906	Ib	OP-1250	CERAN	OP-1250 + ribociclib OP-1250 + alpelisib	Previously treated HR+/HER2− ABC with no more than 2L ET and 1L CT (prior CDK4/6i allowed)	Recruiting
NCT04072952	I/II	ARV-471	PROTAC	ARV-471 ARV-471 + palbociclib	Previously treated (prior CDK4/6i allowed) postmenopausal MBC HR+/HER2−	Recruiting
NCT05501769	Ib	ARV-471	PROTAC	ARV-471 + everolimus	Previously treated HR+/HER2− ABC with prior CDK4/6i	Recruiting
NCT05654623 (VERITAC-2)	III	ARV-471	PROTAC	ARV-471 Fulvestrant	Advanced/MBC HR+/HER2- with progression on ET + CDK4/6i, with at least 6 months of ET prior to PD	Recruiting
NCT05573555 (TACTIVE-U)	Ib/II	ARV-471	PROTAC	ARV-471 + ribociclib	Previously treated HR+/HER2− ABC with prior CDK4/6i, up to 2L prior therapies	Recruiting
NCT05548127 (TACTIVE-U)	Ib/II	ARV-471	PROTAC	ARV-471 + abemaciclib	Previously treated HR+/HER2− ABC with prior CDK4/6i in any setting	Recruiting

*1* *L* 1 line of therapy, *AI* aromatase inhibitor, *CDK4/6i* cyclin-dependent kinase 4/6 inhibitor, *CERAN* complete estrogen receptor antagonist, *CT* chemotherapy, *ET* endocrine therapy, *MBC* metastatic breast cancer, *PROTAC* proteolysis targeting chimera, *SERCA* selective estrogen receptor covalent antagonist, *SERD* selective estrogen receptor degrader, *AE* adverse events.

ARV-471 is a novel PROTAC evaluated in the phase I/II VERITAC study enrolling patients with HR + /HER2− ABC. ARV-471 monotherapy showed a clinical benefit rate (CBR) of 40% in the phase I dose-escalation study in patients who received prior ET and CDK4/6i. Phase II dose expansion confirmed its CBR of 37.1% with 200 mg QD dose and 38.9% with 500 mg QD dose, with the suggestion of enhanced activity in *ESR1*-mutated subgroup^36^. Part C of the study is looking at ARV-471 in combination with palbociclib in pretreated HR + /HER2− ABC and is currently recruiting patients (Clinical Trials.gov identifier NCT04072952), while the ongoing phase 3 VERITAC-2 trial will be comparing ARV-471 against fulvestrant after progression on first-line ET with CDK4/6i (NCT05654623).

Results from randomized trials on several novel SERDs were recently presented. Elacestrant demonstrated a significant progression-free survival (PFS) benefit over physician’s choice of standard-of-care (SOC) ET (fulvestrant or AI) in the open-label randomized phase III EMERALD trial, where patients had progressed after 1–2 lines of ET for HR + /HER2− ABC, including prior CDK4/6i, and maximum of one line of palliative chemotherapy. Primary endpoints of centrally reviewed PFS in all patients (intent-to-treat (ITT) population, *n* = 477) and in patients with detectable *ESR1* mutations from circulating tumor DNA (ctDNA) were met (hazard ratio (HR) 0.70, *P* = 0.002 and HR 0.55, *P* = 0.0005, respectively). The Kaplan–Meier survival curves dipped significantly during the first 3 months in both arms, reflecting the limited benefit from endocrine monotherapy in majority of patients. However, the 12-month PFS achieved with elacestrant in the ITT population was 22.3% versus 9.4% with SOC ET, supporting its activity in endocrine-sensitive tumors. In addition, the magnitude of PFS improvement was higher in patients with detectable *ESR1* mutation^24^. Elacestrant was generally well-tolerated. Nausea of any grade was reported in 35.0% of patients receiving elacestrant compared to 18.8% receiving SOC; grade 3/4 nausea was uncommon at 2.5% and 0.9% respectively. Ocular or cardiac toxicities which have been reported with other SERDs, SERMs or SERCAs, such as giredestrant, camizestrant^37^, tamoxifen^38^, and H3B-6545, were not observed with elacestrant^24,39^.

Camizestrant is a next-generation oral SERD and pure ER antagonist^40^. The phase II SERENA-2 trial reported superiority of camizestrant over fulvestrant in patients with recurrence or progression on 1 line of ET and no more than 1 line of chemotherapy. Investigator-assessed mPFS was 7.2 months with 75 mg camizestrant and 7.7 months with 150 mg dose, compared to 3.7 months with fulvestrant (HRs 0.58, *P* = 0.0124 and 0.67, *P* = 0.0161, respectively)^41^. These positive results contrast with the negative randomized phase II studies of two other oral SERDs, amcenestrant and giredestrant. They did not meet their primary objective of PFS improvement in pretreated HR + /HER2− ABC over ET of physician’s choice^42,43^, although a numerical trend suggested greater benefit in the presence of *ESR1* mutations. A prespecified interim analysis of the phase III AMEERA-5 trial (NCT04478266) comparing first-line amcenestrant and palbociclib with letrozole and palbociclib did not meet the prespecified boundary for continuation in patients with HR + /HER2- ABC, leading to termination of the amcenestrant clinical development program.

While the discordant results may be related to different intrinsic properties influencing the potency of the various compounds^39,44^, patient selection criteria or imbalance of tumor characteristics in small randomized phase 2 trials may also impact on the efficacy results. The benefit of SERD over SOC ET appears to be more pronounced in patients with endocrine-sensitive tumors harboring *ESR1* mutations, without significant activation of other pathways. The Food and Drug Administration (FDA) approved elacestrant for postmenopausal women or adult men with ER + /HER2−, *ESR1*-mutated ABC after progression on at least one line of ET in January 2023, and the Guardant360 CDx assay as a companion diagnostic. As of May 2023, to aid in treatment selection, routine testing for *ESR1* mutations from blood or tumor biopsy at progression, is now recommended based on the updated American Society of Clinical Oncology (ASCO) guidelines^45^.

### Continuing CDK4/6 inhibition beyond progression with switch in ET

In the phase III PADA-1 trial, 172 patients with rising circulating *ESR1* mutation before radiological progression on first-line AI and palbociclib were randomized to continuing their initial treatment or to receive palbociclib with fulvestrant. After median follow-up of 26.0 months from randomization, mPFS from assignment improved from 5.7 months in the control arm to 11.9 months with early switch to fulvestrant (stratified HR = 0.61, *P* = 0.0040)^23^. Data are awaited on whether this “lead-time” PFS advantage from early switch will translate into any meaningful OS benefit, warranting change in clinical practice. SERENA-6 (NCT04964934) is designed to assess the switching to camizestrant versus continuing an AI for HR + /HER2- ABC patients on first-line AI and CDK4/6i with *ESR1* mutation detected on ctDNA without clinical progression.

In BioPER, a single-arm phase II trial of continuing palbociclib with switch to ET of physician’s choice upon progression on prior palbociclib + ET, CBR was 34.4% with mPFS of 2.6 months^46^. Biomarker analysis showed loss of *Rb* in 13% of tumors, which did not benefit from continuing palbociclib. More recently, preliminary results from the randomized phase II PACE trial failed to show PFS improvement in continuing palbociclib with fulvestrant (*n* = 111) over fulvestrant alone (*n* = 55) after progression on AI and CDK4/6i; >90% of patients had received prior palbociclib^47^.

In the phase II MAINTAIN trial, 119 patients whose HR + /HER2− ABC progressed on ET plus any CDK4/6i and ≤1 prior line of chemotherapy were randomized to ribociclib versus placebo with fulvestrant or exemestane. In terms of prior CDK4/6i, 87% had received palbociclib. In the ITT population, mPFS was 5.29 months in the ribociclib arm compared to 2.76 months in the placebo arm (HR 0.57, *P* = 0.006). Results were similar in the fulvestrant subgroup^48^. Taken together, the data seems to show a possible benefit of switching from one CDK4/6i to another, versus re-exposure to the same CDK4/6i upon progression. While these results suggest benefit from ribociclib, a confirmatory phase III trial is essential before this strategy is adopted as routine clinical practice. Moreover, such an approach will not be effective if the tumors have lost expression of ER, developed *RB1* mutation or loss, or are driven by activation of alternate pathways. The tumor biology should be reassessed in each patient with serial biopsies or liquid biopsy if feasible, and provided it may alter clinical management.

### Targeting the androgen receptor pathway

Androgen receptor (AR) is expressed in up to 90% of ER+ BCs^49^. AR expression portends a favorable prognosis with its role as a tumor suppressor by antagonizing ER target genes and repressing expression of cell cycle genes^50^. In a randomized phase II trial, adding enzalutamide, an AR inhibitor, to exemestane failed to improve PFS compared to exemestane alone in patients with 0-1 prior line of endocrine therapy. Low levels of *ESR1* mRNA expression with high AR expression predicted benefit from the addition of enzalutamide in an exploratory analysis^51^. However, a recent study uncovered the differential effects of AR inhibition depending on the AR to ER ratio. In AR-low BC cells, enzalutamide displaced estrogen from ER binding sites, functioning as ER antagonist. In the AR-high setting where AR repressed ERα signaling, enzalutamide antagonized AR, promoting ERα signaling, while RAD140, a selective AR agonist, activated AR signaling and suppressed AR-high tumor growth by inhibiting ER^52^.

Enobosarm, a selective androgen receptor modulator (SARM) which activates AR in breast, muscle and bone without the virilizing side effects associated with steroidal androgens, was assessed in the open-label phase II G200802 trial among patients with AR+ (nuclear staining >10%), ER + ABC. The primary endpoint of CBR at 24 weeks was 32% in the 9 mg group (*n* = 72) and 29% in the 18 mg group (*n* = 64). ORR was 48% in tumors that were AR ≥ 40% positive versus 0% with AR < 40%^53^. Enobosarm was granted fast-track designation by the FDA for the treatment of AR + /HR + /HER2− ABC in January 2022; randomized phase III monotherapy and combination therapy trials are ongoing (NCT04869943, NCT05065411).

### Targeting the PI3K/AKT/mTOR pathway

The PI3K/AKT/mTOR pathway can be activated by *PIK3CA* activating mutations and amplification, loss of tumor suppressors such as *PTEN*, *INPP4B*, and overexpression and/or mutation of upstream receptor tyrosine kinases (RTKs). PI3K/AKT/mTOR signaling promotes cellular proliferation and resistance to apoptosis; AKT can also activate ER-mediated transcription independently of estrogen, leading to endocrine resistance^54,55^. In HR + /HER2− ABC, the prevalence of activating *PIK3CA* mutations is estimated at ~30–40%, and 5–10% for activating *AKT1* mutations and inactivating *PTEN* alterations separately^11,56–59^. *PIK3CA*-mutated HR + /HER2− ABC have also been associated with inferior survival outcomes compared to wild-type tumors^60,61^.

In the SOLAR-1 trial, addition of alpelisib, an alpha-specific PI3K inhibitor—better tolerated than the older generation of pan-PI3K inhibitors, to fulvestrant in the cohort of patients with *PIK3CA*-mutated ABC improved the mPFS from 5.7 months to 11 months (HR 0.65, *P* < 0.001). The 7.9-month numeric improvement in median OS was not statistically significant. No benefit was observed in patients with *PIK3CA*-wild-type tumors. FDA’s approval of alpelisib and fulvestrant in *PIK3CA*-mutated HR + /HER2− ABC, along with the updated ASCO guidelines, recognized *PIK3CA* mutation (tumor or liquid biopsy) as a biomarker to guide systemic therapy in ABC^62,63^. However, merely 6% of subjects had received prior CDK4/6i in the SOLAR-1 trial^58,64^.

BYLieve is a phase II, open-label, three-cohort, non-comparative study of alpelisib plus either fulvestrant or letrozole in patients with HR + /HER2− *PIK3CA* mutant ABC progressing on or after prior treatments, including CDK4/6i (Table 2). All patients in cohorts A and B had received CDK4/6i with AI and fulvestrant, respectively, as the immediate past treatment, whilst 66.7% of those in cohort C received prior CDK4/6i^65–67^. At 6 months, 50.4% of patients in cohort A, 46.1% in cohort B and 48.7% in cohort C were alive and without disease progression. mPFS was slightly lower than that in the predominantly CDK4/6i naive SOLAR-1 study population. The most frequent grade 3/4 adverse events (AEs) included hyperglycemia and rash which can affect dose delivery and efficacy. Frequency of AEs leading to discontinuation of alpelisib was lower compared to in SOLAR-1, possibly due to increasing familiarity and improved management of toxicities with measures such as prophylactic antihistamines for rash. Prophylactic metformin use was able to reduce the severity and incidence of alpelisib-induced hyperglycemia in the METALLICA study^68^. Other challenges include the development of inactivating *PTEN* mutations, adaptive rewiring, epigenetic and metabolic reprogramming, rendering the cancer cells resistant to PI3K inhibition^69,70^. Given the importance of PI3K inhibition, novel PI3K inhibitors which degrade the mutant oncoprotein selectively such as inavolisib (GDC-0077)^71^, RLY-2608 (NCT05216432), and LOXO-783, an allosteric *PIK3CA* H1047R-mutant-specific inhibitor^72^, and may be better tolerated, are being tested in clinical trials currently.

**Table 2:** Targeting PI3K/Akt pathway post CDK4/6 inhibitor: results of phase III SOLAR-1(alpelisib arm), phase II BYLieve and phase III CAPItello-291 (capivasertib arm) trial studies.

	BYLieve Cohort A	BYLieve Cohort B	BYLieve Cohort C	SOLAR-1 Alpelisib arm, *PIK3CA*-mutated cohort	CAPItello-291 Capivasertib arm, ITT population
*N*	127	126	126	169	355
Cohort details	Immediate prior AI + CDK4/6i	Immediate prior fulvestrant + CDK4/6i	Immediate prior CT or ET	Recurrence/progression on/after AI	Prior ET, prior CDK4/6i allowed
Treatment arm	Fulvestrant + alpelisib	Letrozole + alpelisib	Fulvestrant + alpelisib	Fulvestrant + alpelisib	Fulvestrant + capivasertib
Median prior lines of palliative treatment	1	1	2	1	1
Prior CDK4/6i in metastatic setting (%)	100	100	66.7	5.3	69.0
Prior CT in metastatic setting (%)	6.3	Not available	46.0	0	18.3
Prior fulvestrant (%)	0	100	32.5	0	0
Median PFS (months) (95% CI)	7.3 (5.6–8.3)	5.7 (4.5–7.2)	5.6 (5.4–8.1)	11.0 (7.5–14.5)	7.2 (5.5–7.4)
Median OS (months) (95% CI)	17.3 (17.2–20.7)	Not reported	Not reported	39.3 (34.1–44.9)	Not mature
ORR (%) (95% CI)	17 (11–25)	15.7	24.3 (16.8–33.2)	26.6 (20.1–34.0)	22.9
Discontinuation due to AE (%)	20	14.3	15.1	25.4	13
AEs, % (all/≥ grade 3)	99/67	100/69.8	99.2/67.5	99.3/76.0	96.6/41.7
Hyperglycemia	69/29	63.5/25.4	65.1/23.8	63.7/36.6	16.3/2.3
Rash	29/10	31.0/9.5	38.9/13.5	35.6/9.9	38.0/12.1 (all types)
Diarrhea	60/6	67.5/4.0	52.4/3.2	57.7/6.7	72.4/9.3
Nausea	46/0	54.8/2.4	40.5/2.4	44.7/2.5	34.6/0.8
Fatigue	29/1	31.0/4.0	31.1/4.0	24.3/3.5	20.8/0.6
Reference	Rugo et al.^65^	Rugo et al.^66^	Rugo et al.^67^	André et al.^58^ André et al.^64^	Turner et al.^74^

*AI* aromatase inhibitor, *CDK4/6i* cyclin-dependent kinase 4/6 inhibitor, *CT* chemotherapy, *ET* endocrine therapy, *ORR* objective response rate, *OS* overall survival, *PFS* progression-free survival.

The results from the randomized double-blind phase III CAPItello-291 trial which tested the addition of capivasertib, an AKT inhibitor (starting dose of 400 mg twice daily for 4 days, followed by 3 days off), to fulvestrant in 708 patients after progression on ≤2 lines of prior endocrine therapy and ≤1 line of prior chemotherapy appear promising (Table 2). Unlike the randomized Phase II FAKTION trial where the benefit of capivasertib was predominantly in patients with PI3K/AKT/PTEN pathway alterations^59,73^, mPFS was doubled with the addition of capivasertib from 3.6 months to 7.2 months (HR 0.60, two-sided *P* < 0.001) in the overall ITT population, and increased from 3.1 months to 7.3 months (HR 0.50, two-sided *P* < 0.001) in the AKT-pathway altered population^74^. While this suggests that the benefit of capivasertib may not be restricted to tumors harboring *PIK3CA*, *AKT1*, or *PTEN* alteration, the study was not powered to detect the benefit in non-altered tumors. Diarrhea, rather than hyperglycemia, was the most common all-grade AE (72.4% versus 16.3%) despite a less stringent criteria of baseline HbA1c < 8.0%. On the other hand, 38.0% of patients in the capivasertib arm experienced any type of rash, compared to only 7.1% in control arm. The positioning of capivasertib with fulvestrant is currently unclear, without direct comparison of its efficacy with alpelisib or everolimus. Moreover, there is no prospective data on the activity of each agent after prior exposure or resistance to another drug targeting the same pathway. However, capivasertib, with its intermittent dosing schedule, may play an increasing role if it is better tolerated than the current PI3K or mTOR inhibitors, or should there be an overall survival benefit.

In contrast, IPATUNITY130 did not show improved efficacy of addition of ipatasertib, another AKT inhibitor, to paclitaxel in patients with HR + /HER2- *PIK3CA*/*AKT1/PTEN*-altered ABC. This was postulated to be due to lack of endocrine blockade^75^. Results from randomized trials testing the efficacy of adding ipatasertib to fulvestrant are awaited (NCT04650581, NCT04060862).

Although the everolimus trials were conducted in the pre-CDK4/6i era, mTOR inhibition remains a valid treatment option for patients regardless of *PIK3CA* mutation status^2,3^. While alpelisib with ET is often the preferred option post CDK4/6i for *PIK3CA*-mutated tumors, mTORC1 activation by feedback loop limits the sensitivity to alpha-specific PI3K inhibitors^69^. Hence mTOR inhibition may potentially be considered after progression on PI3K inhibitor. The triplet combination of ET with CDK4/6i and PI3K/AKT/mTOR inhibitors was explored in several early-phase trials (Table 3), based on the rationale that PI3K/AKT/mTOR pathway aberrations may confer resistance to CDK4/6i, and preclinical evidence of CDK4/6i re-sensitizing resistant cells to PI3K inhibitors^76^. TRINITI-1 investigated exemestane, everolimus, and ribociclib in patients who progressed on CDK4/6i; the CBR was 41.1% and mPFS was 5.7 months^77^. Although the triplet combination of fulvestrant, ribociclib, and alpelisib was not feasible due to toxicities^78^, the combinations of fulvestrant and palbociclib with ipatasertib, capivasertib or inavolisib continue to be tested in ongoing trials (NCT04060862, NCT04862663, NCT04191499).

**Table 3:** Selected triplet combinations targeting ER, CDK4/6, and PI3K/AKT/mTOR.

	TRINITI-1	IPATUNITY 150	NCT03006172		NCT02684032	
*N*	104	20	36 for arms E and F		103; 59 for arms C and D	
Phase	I/II	Ib/III	I/Ib		Ib (dose expansion)	
Cohort details	HR+/HER2- ABC after progression on CDK4/6i	HR+/HER2- ABC after progression on first-line ET or primary endocrine-resistant	Primary endocrine-resistant, *PIK3CA*-mutated, HR+/HER2− ABC		Previously treated HR+/HER2−ABC; CDK4/6i-pretreated for arms C and D	
Treatment arm	Exemestane + ribociclib + everolimus	Fulvestrant + palbociclib + ipatasertib	Fulvestrant + palbociclib + inavolisib	Fulvestrant + palbociclib + inavolisib + metformin (obese/prediabetic)	Fulvestrant + palbociclib + gedatolisib (weekly)(arm C)	Fulvestrant + palbociclib + gedatolisib (3weeks on, 1 week off)(arm D)
ORR (%) (95% CI)	8.4 (3.7–15.9)	55 (32–77)	40	13	32 (16–52)	63 (42–81)
CBR (%) (95% CI)	41.1 (31.1–51.6)	95	58%		79 (59–92)	96 (81–100)
Median PFS (months) (95% CI)	5.7 (3.6–9.1)	Not mature	Not reported		5.1 (3.4–7.5)	12.9 (7.4–16.7)
AEs, % (All/≥ grade 3)						
Neutropenia	69.2/51.0	75/65	85/65	56/56	66/56	81/67
Stomatitis	40.4/2.9	40/0	80/10	50/0	88/22	89/22
Diarrhea	27.9/1.9	80/15	45/5	50/0	31/6	52/7
Hyperglycemia	18.3/6.7	~20/0	60/15	69/44	25/9	26/7
Reference	Bardia et al.^77^	Oliveira et al.^159^	Bedard et al.^160^		Layman et al.^161^	

*AI* aromatase inhibitor, *CDK4/6i* cyclin-dependent kinase 4/6 inhibitor, *CBR* clinical benefit rate, *DCR* disease control rate, *ET* endocrine therapy, *ORR* objective response rate, *PFS* progression-free survival.

### Targeting the Ras/Raf/MAPK pathway

Alterations in the Ras/Raf/MAPK pathway such as *NF1, KRAS, HRAS, BRAF*, *ERBB2*, and *EGFR*, are enriched in endocrine-resistant HR + /HER2− tumors. They appear to be mutually exclusive with *ESR1* mutations, occurring in 16% of *ESR1*-wild-type BCs post-ET^11^. Increased signaling promoted cellular growth and proliferation, and induced an ER-negative phenotype in vitro^11,79^.

Selumetinib, a MEK1/2 inhibitor, was tested in a randomized phase II trial with fulvestrant against placebo and fulvestrant in HR + /HER2− ABC post 1st line AI. Trial recruitment was halted after the selumetinib combination failed to reach the prespecified disease control rate (DCR) at the interim analysis, faring worse than fulvestrant/placebo with DCR of 23% vs 50%, respectively^80^. Possible reasons include significant toxicities such as mucocutaneous disorders, fatigue, nausea/vomiting, edema, diarrhea impacting dose delivery, compensatory activation of alternative signaling pathways, lack of efficacy in a biomarker-unselected population, and imbalance of other factors due to chance with the small sample size. As such, the role of targeting the MAPK pathway remains to be explored. Current trials are evaluating newer-generation MEK inhibitors in ABC or solid tumors harboring *NF1* or MAPK-activating mutations (NCT05554354, NCT05054374).

### Targeting receptor tyrosine kinases

Overexpression or activating mutation of RTKs such as FGFR and HER2 lead to activation of downstream oncogenic signaling pathways, including the PI3K/AKT/mTOR and RAF/RAS/MAPK pathways. Aberrant FGFR signaling has been shown to mediate resistance to ET, CDK4/6, and PI3K inhibitors. PFS was shorter in patients with *FGFR1* amplification on ET and CDK4/6i^81,82^. FGFR axis alterations such as *FGFR1*, *FGFR2*, or *FGF3* can also be acquired, detected in up to around 40% of resistant specimens^12,81,83^.

Phase II trials of FGFR inhibitors dovitinib^84^, lucitanib^85^, and AZD4547^86^ in unselected patients showed modest activity, and were complicated by toxicities such as hypertension, hypothyroidism and retinal detachments. The benefit appeared to be restricted to tumors with *FGFR1* amplification or overexpression, or dependence on FGFR signaling. In the preliminary report of a phase Ib trial testing fulvestrant, palbociclib and erdafitinib, a pan-FGFR inhibitor in *FGFR*-amplified/ER + /HER2-negative pretreated ABC, toxicities led to treatment discontinuation in several patients. No objective responses were observed, though PFS was longer (6 months) in patients with high levels of *FGFR1* amplification or *FGFR3* amplification^87^.

Lenvatinib, a multi-kinase inhibitor with activity against VEGFR1-3 (vascular endothelial growth factor receptor), FGFR1-4, RET (rearranged during transfection), PDGFR (platelet-derived growth factor receptor) and KIT, was investigated with letrozole in a phase Ib/II trial. ORR was 23.3% in 31 patients who had received median 5 lines of prior therapy, with median duration of response lasting 6.9 months^88^. RET expression levels on immunohistochemistry were not predictive of response; further biomarker analyses are awaited.

The prevalence of activating *HER2* mutations varies from 2 to 4% in primary BCs to >5% in lobular cancers and metastatic lesions^89–91^. These mutations may span across the extracellular, transmembrane or juxta-membrane domain^90^. Neratinib, an irreversible pan-HER inhibitor, inhibited cell growth^90^ and restored sensitivity to fulvestrant in estrogen-resistant HER2-mutant cancer cells in vitro^91^.

In the phase II MutHER study, combination of neratinib and fulvestrant achieved CBR of 38% and 30%, respectively, in the fulvestrant-treated and fulvestrant-naive patients with HR + /HER2− *HER2*-mutated ABC^92^. Upon progression, addition of trastuzumab was further able to induce a partial response in 3 out of 5 patients, supporting role of dual-HER2 blockade in overcoming resistance to neratinib^92^. The SUMMIT trial included 33 patients with HR + /HER2− *HER2*-mutated ABC on fulvestrant, trastuzumab and neratinib; ORR was 42.4% and mPFS was 7.0 months^93^. HER2-directed antibody–drug conjugates (ADC) may be useful, but the indication in HR + /HER2− ABC is for HER2-low (details below), unlike lung cancer where FDA approval is for *HER2*-mutated non-small cell lung cancer^94^.

### Targeting cancer epithelial antigens via antibody–drug conjugates (ADCs)

Beyond targeting pathways, novel ways of delivering chemotherapy have afforded new therapeutic options in the endocrine-resistant setting. ADCs allow the delivery of cytotoxic drugs directly to cancer cells via targeting specific cell-surface antigens. Novel ADCs with enhanced properties such as higher payload to antibody ratio, stable tumor-selective cleavable linker and potent, membrane-permeable payload with short systemic half-life and bystander killing effect have achieved breakthroughs in the cancer treatment landscape^95^.

The positive results of the phase III DESTINY-Breast04 led to FDA approval of trastuzumab deruxtecan (T-DXd) in August 2022 for treatment of HER2-low ABC in patients who have received prior chemotherapy in the metastatic setting or developed disease recurrence during or within six months of completing adjuvant chemotherapy^96^. T-DXd contains a novel topoisomerase I inhibitor payload, which is seldom used in ABC, with a drug-to-antibody ratio of 8:1^97^. HER2-low BC, defined by immunohistochemical scoring of IHC 1+ or 2+ with negative in situ hybridization (ISH), accounts for ~60% of HER2- ABC^98^. HER2-low BCs do not respond to traditional HER2-targeted therapy and are classified as HER2 negative. However, novel HER2-directed ADCs can deliver the cytotoxic payload to the surrounding tumor cells through the bystander effect^95^.

DESTINY-Breast04 randomized 557 patients with HER2-low ABC in a 2:1 ratio to T-DXd versus the physician’s choice of chemotherapy. Subjects with HR + /HER2-low ABC (*n* = 494) had received a median of 3 lines of prior palliative therapy, including 1–2 lines of palliative chemotherapy, with prior CDK4/6i in approximately 70% of the cohort (Table 4). The primary endpoint of PFS in the HR + /HER2- cohort was significantly superior with T-DXd compared to control: median 10.1 months versus 5.4 months (HR 0.51, *P* < 0.001), with OS improvement as well (median 23.9 months versus 17.9 months, HR = 0.64, *P* = 0.003). Drug-related interstitial lung disease or pneumonitis, an AE of special interest, was observed in 12.1% of patients; 0.8% were grade 5 events, underscoring the need for close surveillance and prompt management.

**Table 4:** Reported trials of antibody–drug conjugates in HR + /HER2− advanced breast cancers.

	DESTINY-Breast04	TROPiCS-02	U31402-A-J101
Phase	III	III	I/II
*N* (patients with HR+HER2- ABC receiving ADC)	331	272	113
Experimental arm	Trastuzumab deruxtecan 5.4 mg/kg IV q21d	Sacituzumab govitecan 10 mg/kg IV D1,8 q21d	Patritumab deruxtecan IV q21d
Median lines of prior therapy (range)	3 (1–9) lines of prior palliative therapy	7 (3–17) lines of therapy in non-metastatic and metastatic setting 3 (0–8) CT in metastatic setting	7 (2–14) lines of therapy in non-metastatic and metastatic setting 3 (1–7) CT in advanced setting
Prior CDK4/6i in metastatic setting (%)	70.4	100	Not reported
Median PFS (months)	10.1 vs 5.4	5.5 vs 4.0	7.4
Hazard ratio	0.51 (95% CI, 0.40–0.64)	0.66 (95% CI, 0.53–0.83)	
Median OS (months)	23.9 vs 17.5	14.4 vs 11.2	14.6
Hazard ratio	0.64 (95% CI, 0.48–0.86)	0.79 (95% CI, 0.65–0.96)	
ORR (%)	52.6 vs 16.3	21 vs 14, *P* = 0.03	30.1
Treatment related Grade ≥3 AEs (%) in ADC arm	52.6*	74	65.9*
Neutropenia (Grade ≥3)	13.7*	51	27.1–52.0*
Thrombocytopenia (Grade ≥3)	5.1*	Not available as only reported AEs ≥5% frequency	27.1–38.8*
Anemia (Grade ≥3)	8.1*	6	20.8–21.4*
Diarrhea (Grade ≥3)	1.1*	9	3.1–4.2*
AEs leading to discontinuation (%) in ADC arm	16.2*	6	9.9*
Interstitial lung disease (%) in ADC arm	12.1 (all grades) 1.3 grade 3–4, 0.8 grade 5*	0	6.6* (all grades)
Reference	Modi et al.^96^	Rugo et al.^110^; Rugo et al.^111^	Krop et al.^107^

*AE* adverse events, *IV* intravenous, *ORR* objective response rate, *OS* overall survival, *PFS* progression-free survival, q21d every 21 days, *TPC* treatment of physician’s choice.

*Values for overall study population—HR+ and HR- in DESTINY-Breast04; all doses and all arms/subgroups in U31402-A-J101.

DAISY, an open-label phase II trial, evaluated the efficacy of single-agent T-DXd with extensive biomarker analysis in 3 cohorts of ABC patients. The primary endpoint of ORR was 70.6% in Cohort 1 (HER2 overexpressing), 37.5% in Cohort 2 (HER2-low) and 29.7% in cohort 3 (HER2-null: IHC 0). Although clinically meaningful activity was seen in HER2-null BC, the mPFS was 4.2 months in Cohort 3, compared to 6.7 months in HER2-low and 11.1 months in HER2-overexpressing cohorts. T-DXd antitumor activity was associated with levels of HER2 expression although other mechanisms could be involved^99^. The activity of T-DXd in HER2-null in this trial and the interobserver reproducibility of HER2 immunohistochemical scoring^100,101^ underscore the limitations of the current HER2-low definition based on immunohistochemical testing. Quantitative HER2 assays with greater sensitivity for lower range of HER2 expression have been suggested as a means to better predict response to T-DXd^102,103^.

HER3 is another RTK belonging to the HER family. Whilst HER3 is not oncogenic when expressed alone, it can be activated through the formation of heterodimers with other receptors and members of the EGFR, effecting downstream PI3K/AKT-pathway signaling. HER3 expression is associated with disease progression and increased metastatic rate. In one study, 30% of primary BCs expressed HER3, but the expression increased to 60% of the matched metastatic specimens^104,105^. Patritumab deruxtecan (HER3-DXd), a novel ADC against HER3, exhibited cytotoxic activity in vitro via HER3-specific binding to cancer cells and release of its topoisomerase payload intracellularly^106^. In the U31402-A-J101 phase I/II study of HER3-DXd in HER3-expressing ABC, ORR was 30.1% with mPFS of 7.4 months in the HR + /HER2− cohort, despite being heavily pretreated (Table 4)^107^.

TROP2, a transmembrane calcium signal transducer, is highly expressed in TNBC and HR + /HER2- BC cells, with prevalence exceeding 90%^108^. Sacituzumab govitecan (SG), an anti-TROP2 (trophoblast cell-surface antigen 2) ADC with a topoisomerase inhibitor payload (drug-to-antibody ratio 8:1), was first tested in triple-negative BC (TNBC)^109^. The subsequent phase III TROPiCS-02 trial in HR + /HER2- ABC showed PFS and OS improvement with SG over the physician’s choice of chemotherapy (mPFS 5.5 months vs 4.0 months; HR 0.66, *P* = 0.0003; mOS 14.4 months vs 11.2 months; HR 0.79, *P* = 0.02). While the benefit of SG may seem less impressive compared to T-DXd in DESTINY-Breast04, the TROPiCS-02 trial population was more heavily pretreated (Table 4)^110,111^. Although PFS and OS favored SG over TPC across TROP2 expression levels (H-score <100 and ≥100), including those with H-score ≤10, only 17% of the trial population showed very low TROP2 expression^112^. In February 2023, FDA-approved SG for patients with HR + /HER2− ABC who have received endocrine-based therapy and at least two additional systemic therapies in the metastatic setting. Datopotamab–deruxtecan (Dato-DXd)—an ADC targeting TROP2, with the same cytotoxic payload as T-DXd, showed an ORR of 29% and a DCR of 85% in a heavily pretreated HR + /HER2− ABC population (median 5 prior lines of treatment) in the phase I TROPION-PanTumor01 trial^113^. The phase III TROPION-Breast01 (NCT05104866) is currently recruiting patients with HR + /HER2− ABC after 1–2 lines of prior chemotherapy.

Table 5 outlines the ongoing trials of ADCs being evaluated for HR + /HER2− or HER2-low ABC. While ADCs appear promising, different ADCs vary considerably in terms of potency and toxicity profiles. Many questions remain to be explored—the optimal predictive biomarker, the sequencing of one ADC after another, the mechanisms of resistance and the cross-resistance between ADCs with similar target antigen or payload, among others. In view of the promising activity, phase 3 trials comparing T-DXd or SG with chemotherapy of the physician’s choice in the first-line chemotherapy setting are ongoing. Apart from improvements in survival outcomes, the impact on cumulative toxicities and quality of life (QOL) will be important, especially for less heavily pretreated patients, who will be receiving the treatment over a longer period of time. Cost-effectiveness, financial toxicities and inequities in access should also be considered.

**Table 5:** Selected trials of antibody–drug conjugates recruiting patients with HR + /HER2− or HR + /HER2-low advanced breast cancers—updated (August 2023).

Clinicaltrials.gov identifier	Phase	Antibody–drug conjugate	Drug target	Treatment arms	Study population	Study status
NCT04494425 (DESTINY-Breast06)	III	Trastuzumab deruxtecan	HER2	Trastuzumab deruxtecan vs TPC (capecitabine, paclitaxel, nab-paclitaxel)	Advanced/metastatic HR+/HER2-low breast cancer with at least 2 prior ET or PD within 6 months of first-line ET + CDK4/6i but no prior CT	Active, not recruiting
NCT04556773 (DESTINY-Breast08)	Ib	Trastuzumab deruxtecan	HER2	Trastuzumab deruxtecan + capecitabine or durvalumab + paclitaxel or capivasertib or anastrozole or fulvestrant	Part 1: previously treated HER2-low advanced/metastatic breast cancer Part 2: HER2-low MBC previously untreated or only 1 prior line	Active, not recruiting
NCT04042701 (KEYNOTE 797)	Ib	Trastuzumab deruxtecan	HER2	Trastuzumab deruxtecan + pembrolizumab	Advanced/metastatic breast cancer, HER2-low with prior failed standard treatments or HER2+ with prior ado-trastuzumab emtansine	Recruiting
NCT04699630	II	Patritumab deruxtecan	HER3	Patritumab deruxtecan	Advanced/metastatic breast cancer—includes HER2+, HR+/HER2-low and TNBC	Recruiting
NCT04965766 (ICARUS-BREAST)	II	Patritumab deruxtecan	HER3	Patritumab deruxtecan	Advanced/metastatic HER3 high, HR+/HER2− breast cancer resistant to ET + CDK4/6i	Recruiting
NCT04639986 (EVER-132-002)	III	Sacituzumab govitecan	TROP2	Sacituzumab govitecan vs TPC (capecitabine, eribulin, gemcitabine, vinorelbine)	Asian patients with HR+/HER2− MBC with ≥2 but ≤4 prior lines of CT	Active, not recruiting
NCT04448886	II	Sacituzumab govitecan	TROP2	Sacituzumab govitecan +/− pembrolizumab	Advanced/metastatic HR+/HER2− breast cancer PD on/within 12 months of adjuvant ET or with ≥1 prior ET	Recruiting
NCT05143229 (ASSET)	I	Sacituzumab govitecan	TROP2	Sacituzumab govitecan + alpelisib	Previously treated HER2- locally recurrent/metastatic breast cancer	Recruiting
NCT04647916 (SWOG 2007)	II	Sacituzumab govitecan	TROP2	Sacituzumab govitecan	HER2- MBC with CNS progression	Recruiting
NCT05104866 (TROPION-Breast01)	III	Datopotamab deruxtecan	TROP2	Datopotamab deruxtecan vs TPC (capecitabine, eribulin, gemcitabine, vinorelbine)	Advanced/metastatic HR+/HER2− breast cancer with PD on ET and 1–2 lines of CT	Active, not recruiting

*CDK4/6i* cyclin-dependent kinase 4/6 inhibitor, *CNS* central nervous system, *CT* chemotherapy, *ET* endocrine therapy, *MBC* metastatic breast cancer, *PD* disease progression, *TNBC* triple-negative breast cancer, *TPC* treatment of physician’s choice.

### Targeting the homologous recombination repair pathway

*BRCA1/2* tumor suppressor genes are involved in the homologous recombination repair (HRR) pathway. HR+ BCs arising in germline *BRCA* (g*BRCA*) mutation carriers frequently exhibit genomic instability with higher histological grade and Oncotype Dx recurrence score than sporadic tumors^114^. g*BRCA* pathogenic variants were also less likely to co-occur with *PIK3CA* somatic mutations in a recent study^115^, suggesting distinct tumor biology. Real-world studies have reported inferior PFS and OS on ET with CDK4/6i in patients with germline pathogenic variants in DNA repair genes^116,117^, reflecting an area of unmet treatment need.

PARP inhibition in cells with homologous recombination deficiency leads to synthetic lethality. Olaparib and talazoparib are approved for use in patients with *gBRCA* mutations and HER2- ABC in the post-chemotherapy setting following the OlympiAD^118^ and EMBRCA^119^ trials which demonstrated PFS benefit over physician’s choice of chemotherapy. HR + /HER2− patients accounted for around half of the population in both trials; absolute mPFS improvement was around 3 months with HR 0.54–0.58 over TPC, albeit without OS benefit.

In the phase II TBCRC048 trial, 54 patients with ABC and germline or somatic mutations in HRR pathway genes other than g*BRCA* mutations (such as *PALB2*, somatic *BRCA1/2, ATM*, or *CHEK2*) received olaparib^120^. Out of 19 HR + /HER2− patients who had received prior CDK4/6i, 58% achieved a response. Activity was also reported in the smaller Talazoparib Beyond BRCA trial^121^. An ongoing phase II trial (NCT03990896) is recruiting patients with HR + /HER2− and triple-negative ABC with somatic *BRCA1/2* mutations to investigate the effectiveness of talazoparib.

Mechanisms of resistance to PARP inhibitors include target-related effects (e.g., mutations in PARP, upregulation of drug efflux pumps), reversion mutations restoring *BRCA*-dependent homologous recombination, and loss of DNA end-protection and restoration of replication fork stability^122^. The latest research efforts are focusing on the next generation of selective PARP1 inhibitors which may be less myelotoxic, other agents targeting the DNA repair pathway, synergistic combinations and identification of predictive biomarkers beyond g*BRCA* mutations.

### Targeting other components of the cell cycle and cell death

Cyclin E1 (CCNE1) drives the progression of cells from G1 into S and M phases of the cell cycle by binding to and activating cyclin-dependent kinase 2 (CDK2). High CCNE1 mRNA expression was associated with relative resistance to palbociclib in the exploratory biomarker analysis of PALOMA 3 with shorter PFS, and poorer anti-proliferative activity in the Preoperative Palbociclib (POP) trial^123^. Activation of the *MYC* oncogene has also been identified as a mechanism of resistance to palbociclib via compensatory CDK2 activation; CDK2 inhibition suppressed cellular proliferation in vitro^124^. BLU222, a selective CDK2 inhibitor, showed sustained antitumor activity in combination with ribociclib in in vivo HR + /HER2− BC models^125^. Early-phase trials on BLU222 and PF3600 (CDK2/4/6i) in advanced solid tumors including HR + /HER2− ABC are ongoing (NCT05252416, NCT03519178).

Sensitivity of cell lines with overactivation of CDK2 in S phase to checkpoint kinase 1 (CHK1) inhibition has been reported. CHK1 is activated during DNA damage, and facilitates cell cycle arrest to permit DNA damage repair; CHK1 inhibition thus allows sensitive cells to accumulate DNA breaks, leading to cytotoxicity^126^. To our knowledge, there is currently no clinical data in HR + /HER2− BC.

CDK7 is integral in (i) initiation and regulation of gene transcription via activation of RNA polymerase II, (ii) regulating the activity of transcription factors including AR and ER, and (iii) directing cell cycle progression via phosphorylation of other CDKs^127^. Samuraciclib (ICEC0942), a CDK7 inhibitor, induced cell cycle arrest and apoptosis in a preclinical study^128^. Subsequently, samuraciclib with fulvestrant showed a 24-week CBR of 36% in a single-arm study of 31 patients with HR + /HER2− ABC post progression on AI plus CDK4/6i, leading to FDA granting fast-track status for the compound^129^. Interestingly, tumors with *TP53* mutation have a significantly shorter mPFS than tumors with wild-type *TP53* (HR 0.17, *P* = 0.0008), consistent with findings that *TP53* activation is essential to induce transcriptional dependency, rendering the cancer cells susceptible to CDK7 inhibition^130^. Hence there may be limitations with CDK7 inhibitors in aggressive luminal tumors that harbor *TP53* alterations.

Aurora-A kinase (AURKA) is a serine/threonine kinase important for the G2/mitosis transition, regulating centrosome function and mitotic spindle assembly^131^. Amplification or overexpression of AURKA enhances its role in tumorigenesis, and potentially serves as an antitumor target^12^. AURKA inhibition mediated mitotic arrest and apoptosis, exhibiting synthetic lethality with *RB1* loss in vitro and in vivo^12,132^. In an older phase I/II trial, alisertib, an AURKA inhibitor, demonstrated 23% ORR with mPFS of 7.9 months in 26 patients with HR + /HER2− ABC^133^. The addition of alisertib to weekly paclitaxel improved mPFS from 7.1 months with paclitaxel alone to 10.2 months (HR 0.56, *P* = 0.005) in the HR + /HER2− ABC cohort of a randomized phase II trial^134^. However, grade 3/4 stomatitis or myelosuppression limited the tolerability of the combination^134^. A recent randomized phase II trial on 91 patients with prior CDK4/6i and ET showed that addition of fulvestrant to alisertib did not increase the ORR (19.6% in monotherapy arm versus 20.0% in combination arm). Nevertheless, the treatment was well-tolerated and the activity of alisertib monotherapy was considered promising in this pretreated population^135^.

Induction of apoptosis is another potential strategy, given that BCL2, an estrogen-responsive anti-apoptosis protein, is overexpressed in 85% of HR + /HER2− BC^136^. Preclinical models of BCL2 expressing HR + /HER2− cancer cells shows enhanced apoptosis with the use of a BCL2 inhibitor ABT-199 (venetoclax) in combination with tamoxifen^136^, as well as triple therapy of venetoclax with fulvestrant and palbociclib^137^. However, while the activity of venetoclax with tamoxifen appeared promising in a phase 1B trial with an ORR of 54% and CBR 75% for 24 patients with ER + BCL2 + ABC treated at RP2D^138^; the addition of venetoclax to fulvestrant in the randomized phase II VERONICA trial failed to show CBR or PFS benefit over fulvestrant alone in HR + /HER2− ABC post CDK4/6i^139^. The increased BCLXL expression observed post CDK4/6i may have resulted in reduced dependency on BCL2 to evade apoptosis^139^; targeting BCLXL alone or in combination with BCL2 may merit further investigation.

The spindle assembly checkpoint kinase TTK, also known as monopolar spindle 1(MPS1), is a key regulator of chromosomal segregation. CDK4/6i-resistant ER + ABC cells with *RB1* loss harbor mitotic defects and are hypersensitive to TTK inhibitor CFI-402257, with induction of premature chromosome segregation, DNA damage, and cell death^140^. CFI-402257 is being tested in a phase I/II study, with an HR + /HER2− ABC cohort receiving combination with fulvestrant post CDK4/6i (NCT05251714).

### Immunotherapy

While immune checkpoint inhibitors (ICIs) have demonstrated efficacy and transformed the treatment paradigms in TNBC, the efficacy of single-agent PD-1 or PD-L1 blockade in HR + /HER2− ABC has been limited^141,142^. Possible reasons include the lower levels of tumor infiltrating lymphocytes (TILs)^143^, lower tumor mutational burden (TMB) and lower frequency of programmed death-ligand 1 (PD-L1) expression. Biomarkers predicting for response to immunotherapy include positive PD-L1 status^142^, high TMB^144^, APOBEC mutational signatures which have been associated with less favorable responses to ET with CDK4/6i^145,146^, and mismatch repair deficiency which is observed in only 1% of BCs^147^.

Combining immunotherapy with other agents such as ET, small molecule inhibitors, chemotherapy or ADCs have the potential to augment tumor response to immunotherapy. Major challenges remain in overcoming resistance to immunotherapy. In preclinical studies of HR + BC, abemaciclib was able to increase tumor immunogenicity with evidence of suppression of regulatory T-cell proliferation, increased expression of antigen presentation genes, and synergism with PD-1 blockade^148^. However, the high rates of toxicities such as grade ≥3 transaminitis have prevented further development of such combinations^149–151^. Other promising preclinical data using histone deacetylase inhibitors as a priming modulator for immunotherapy^152^ also did not translate into meaningful clinical efficacy when used in combination with ICI^153^.

Although chemotherapy has the potential to stimulate cytotoxic T-cell activation and deplete regulatory T cells with induction of cell death and release of tumor-related antigens^154^, a phase II randomized trial investigating the addition of pembrolizumab to eribulin compared to eribulin alone in HR + /HER2− ABC did not improve PFS, ORR or OS^155^. A randomized phase III trial is currently investigating the addition of pembrolizumab to investigator’s choice of chemotherapy in patients with PD-L1 CPS-positive, CDK4/6i-resistant HR + /HER2− ABC who have not received prior chemotherapy (NCT04895358), presumably with less immune exhaustion.

## Discussion: future directions

In summary, while various systemic therapeutic strategies have emerged in the post-CDK4/6i era, HR + /HER2− ABC remains incurable. There is a pressing need to improve our understanding of resistance mechanisms to not only ET and CDK4/6i, but also other agents discussed in this review. With the loss of commercial interest in developing certain compounds after failure to meet the clinical efficacy endpoint(s), efforts to interrogate the discordance between preclinical and clinical activity have stalled, missing the opportunity to elucidate the biological underpinnings. Treatment resistance is often complicated by dynamic adaptive changes, tumor heterogeneity and toxicities affecting dose delivery in the clinical setting. Loss of ER expression and change in HER2 expression may also occur^12,156,157^. Given the diverse mechanisms of resistance, a one-size-fits-all approach may not always be appropriate. Tumor profiling upon progression may help to characterize the latest biology, and assist in the development of novel therapies to circumvent the various mechanisms of resistance in the optimal sequence (Fig. 2).

Optimal sequencing of treatment options depends on (1) the presence of specific molecular aberrations at the specific timepoint, such as acquired *ESR1* mutations, MAP kinase pathway alterations; (2) the comparative efficacy of selected treatment relative to current gold standard treatment paradigms; (3) the setting for which clinical efficacy of specific treatment is proven in adequately powered clinical trial(s); and (4) the balance between maximizing patient-derived survival benefits versus QOL, financial and other toxicities, when compared to alternative therapy options in the patient’s overall breast cancer treatment journey. While earlier introduction of a highly efficacious drug may prolong PFS and OS to a greater extent, the impact on long-term toxicities, including financial burden, cost-effectiveness and QOL, should be considered.

The SAFIR02-BREAST trial examined the maintenance strategy of matched targeted therapies to genomic alterations versus standard-of-care chemotherapy in patients with HER2- ABC. Although PFS improvement was observed with matched targeted therapies only for genomic alterations classified as level I/II according to the ESMO Scale for Clinical Actionability of Molecular Targets (ESCAT)^158^, this may change with the rapidly evolving treatment landscape. *ESR1* mutation testing at recurrence or progression on ET in HR + /HER2− ABC has now been incorporated into the latest ASCO guidelines^45^, and evaluation of other tumor genomic mutations with the option of ctDNA testing may be increasingly adopted as biomarkers become prospectively validated in clinical studies^62,45^. With the increasing discovery of molecular resistance drivers, the development of novel therapeutics will need to be accelerated, ideally with greater inclusion and diversity in clinical trials. While preliminary results from some early-phase studies may seem promising, adequately powered randomized clinical trials will generally still be required to confirm the efficacy over current standard of care to transform our clinical practice. Patients with resistant or refractory disease continue to be under-represented in industry-sponsored registration trials which focus on less heavily pretreated patients with better prognosis for achieving the primary endpoints. Moving forward, concerted efforts to design and conduct academic trials are instrumental for addressing the unmet needs of various challenging patient subpopulations and dissecting complex biology.

## Conclusion

Currently, ET with CDK4/6i is the standard of care for most patients with HR + /HER2− MBC in the first-line setting, though the treatment paradigm for patients post relapse on adjuvant CDK4/6i is unclear. Based on our understanding of the diverse mechanisms of resistance post tumor progression on ET and CDK4/6i, a personalized rather than one-size-fits-all approach will be the optimal strategy. Molecular profiling at time of progression helps to elucidate the specific molecular aberrations and activated pathways, allowing for better tailoring of systemic therapy. Ongoing efforts to identify new therapeutic targets with predictive biomarkers and development of novel therapies will continue to shape the treatment paradigm in HR + /HER2− MBC.

### Reporting summary

Further information on research design is available in the Nature Research Reporting Summary linked to this article.

### Supplementary information

Reporting summary
